# Identification and Phenotypic Characterization of *ZEBRA LEAF16* Encoding a β-Hydroxyacyl-ACP Dehydratase in Rice

**DOI:** 10.3389/fpls.2018.00782

**Published:** 2018-06-12

**Authors:** Ziwen Liu, Zhiyuan Wang, Han Gu, Jia You, Manman Hu, Yujun Zhang, Ze Zhu, Yihua Wang, Shijia Liu, Liangming Chen, Xi Liu, Yunlu Tian, Shirong Zhou, Ling Jiang, Linglong Liu, Jianmin Wan

**Affiliations:** ^1^State Key Laboratory for Crop Genetics and Germplasm Enhancement, Jiangsu Plant Gene Engineering Research Center, Nanjing Agricultural University, Nanjing, China; ^2^National Key Facility for Crop Gene Resources and Genetic Improvement, Institute of Crop Science, Chinese Academy of Agricultural Sciences, Beijing, China

**Keywords:** *ZEBRA LEAF 16*, β-hydroxyacyl-ACP dehydratase, chloroplast development, fatty acid, *Oryza sativa* L.

## Abstract

The chloroplast is a self-independent organelle and contains its own transcription and translation systems. The establishment of genetic systems is vital for normal plant growth and development. We isolated a rice *zebra leaf 16* (*zl16*) mutant derived from rice cultivar 9311. The *zl16* mutant showed chlorotic abnormalities in the transverse sectors of the young leaves of seedlings. The use of transmission electron microscopy (TEM) demonstrated that dramatic defects occurred in variegated *zl16* leaves during the early development of a chloroplast. Map-based cloning revealed that *ZL16* encodes a β-hydroxyacyl-ACP dehydratase (HAD) involved in *de novo* fatty acid synthesis. Compared with the wild type, a missense mutation (Arg164Trp) in the *zl16* mutant was identified, which significantly reduced enzymatic activity and altered the three-dimensional modeling structure of the putative protein. *ZL16* was ubiquitously expressed in various plant organs, with a pronounced level in the young leaf. A subcellular localization experiment indicated that ZL16 was targeted in the chloroplast. Furthermore, we analyzed the expression of some nuclear genes involved in chloroplast development, and found they were altered in the *zl16* mutant. RNA-Seq analysis indicated that some genes related to cell membrane constituents were downregulated in the mutant. An *in vivo* metabolic assay revealed that the total fatty acid content in the mutant was significantly decreased relative to the wild type. Our results indicate that HAD is essential for the development of chloroplasts by regulating the synthesis of fatty acids in rice.

## Introduction

A chloroplast is a plant organelle that originated from an endosymbiotic event between a photosynthetic cyanobacteria and its eukaryotic host (Moreira et al., 2000; Kusumi et al., 2004). Its main function is to conduct photosynthesis and convert the energy from solar irradiation into chemical energy. Thus, the normal formation and development of chloroplasts is vital to sustain crop yield. Many studies have been conducted to uncover the molecular mechanisms of chloroplast development (Barkan et al., 1995; de Souza et al., 2016).

In addition to being a photosynthetic organelle, the plastid is also a major synthetic site for fatty acids and many amino acids in plants. In plant plastids, the *de novo* biosynthesis of fatty acids is mainly catalyzed by several conserved enzymes, including acetyl-CoA carboxylase (ACCase), malonyl-CoA:ACP transacylase (MAT), β-ketoacyl- ACP synthase (KAS), β-ketoacyl-ACP reductase (KAR), HAD, and enoyl-ACP reductase (ENR) (Gardner et al., 2002).

HAD conducts the third step in the fatty acid synthesis in a plant fatty acid synthase complex (González-Thuillier et al., 2016). Previous studies of *Escherichia coli* determined that HAD possesses two isozymes, viz., β-hydroxydecanoyl-ACP dehydratase/isomerase (FabA) and HAD (FabZ) (Mohan et al., 1994; Heath and Rock, 1996). FabA produces unsaturated fatty acids and is only found in gram-negative bacteria, while FabZ allows the synthesis of saturated as well as unsaturated fatty acids and functions ubiquitously in both gram-positive and negative bacteria. Unlike the dehydratase/isomerase bifunctional enzyme activity of FabA, FabZ only has a dehydration function. Additionally, FabZ is more active than FabA for catalyzing short-chain primer substrates (Mohan et al., 1994; Pillai et al., 2003; Sharma et al., 2003). Because bacteria and plants share a low sequence similarity, sequence-based searching for plant HAD with *E. coli*. HAD homologs was difficult until the recent characterization of a mitochondrial HAD (mtHAD) in *Arabidopsis*. RNA interference (RNAi) mutants, with reduced mtHAD expression, exhibit traits such as a significantly reduced size of aerial organs, photorespiratory deficiency, altered chloroplastic starch granule morphology, and reduced catalytic activity of lipoylated enzymes (Guan et al., 2017). Despite the clarification of the various HADs in the fatty acid biosynthesis of *E. coli* and *Arabidopsis*, the roles of HAD proteins in rice (*Oryza sativa*), a staple crop for over half of the world’s population and a monocot model plant, remain uncertain.

In this study, we identified a rice chlorophyll deficient mutant (*zebra leaf 16*, *zl16*) that develops zebra leaves with reduced chlorophyll contents and abnormal chloroplast microstructures during the early leaf developmental stage. Map-based cloning showed that *ZL16* encodes a FabA-like HAD, targeting the chloroplast. An expression experiment revealed that the *zl16* mutation was likely involved in an alteration of the transcriptional levels of many of the nuclear- and plastid-encoded genes related to chloroplast development. Our results indicated that fatty acid synthesis is closely related to chloroplast development in rice.

## Materials and Methods

### Plant Materials and Growth Conditions

The *zl16* mutant was selected from an ethyl methyl sulfonate (EMS)-induced mutant pool of *indica* c.v. 93-11. F_2_ populations derived from a crossing between *zl16* and 02428 or Wuyunjing 7 were used for genetic analysis. All plants were grown in a paddy field at Nanjing, China (32°N latitude) unless otherwise stated. For a shading treatment, plants were germinated and kept in a growth chamber (ATC40, Conviron, MB, Canada) under a 12:12 h light (440 μmol m^-2^ s^-1^)/darkness cycle at temperatures of 30°C (day)/20°C (night). For temperature treatments, the chamber containing plants was set at 12:12 h light/darkness cycle at a constant temperature of 20°C or 30°C. The genetic materials generated in this study will be provided in a timely manner for non-profit use.

### Quantitative Analysis of Chlorophyll Content

According to a previous study (Lichtenthaler, 1987), pigments from 8 and 12 d seedlings of the 93-11 and *zl16* mutants were extracted. Equal weights of fresh leaves (approximately 30 mg fresh weight) were cut into small pieces, and then immersed in 5 ml of 95% ethanol in darkness, with intermittent shocking. After a 48-h treatment, the absorbance of the supernatants was measured by a spectrophotometer (SpectraMax M3, Molecular Devices, San Jose, CA, United States) at 470, 649, and 665 nm, respectively.

### Transmission Electron Microscopy (TEM) Analysis

To observe the structure of chloroplasts, seeds were soaked in tap water for 2 days and then germinated in a growth chamber. The temperature was set for 16 h at 30°C in the light (photon flux 440 μmol m^-2^ s) and 8 h at 20°C in the dark. The second leaves from 8 and 12-day-old seedlings were harvested. The samples were processed and sectioned according to a method used previously (Wang et al., 2017), and the sections were observed by TEM (H7650, Hitachi, Tokyo, Japan).

### Fine-Mapping and Cloning of *zl16* Locus

An F_2_ population derived from a cross between *zl16* (in the *indica* background) and 02428 (in the *japonica* background) was used for the map-based cloning of *zl16*. Seedlings with the typical mutated phenotype were collected for DNA extraction. Initially, 10 individuals were used for preliminary linkage analysis. Then 944 additional individuals were used to delimit the *zl16* locus to a region flanked by the markers VF3 and VF4. Within this region, the gene divergences between 93-11 and *zl16* were examined by sequencing genomic DNA. Simple sequence repeat (SSR), insert-deletion (In-Del), and derived cleaved amplified polymorphic sequences (dCAPS) markers were developed to confirm the *ZL16* locus. The sequences of the newly developed primers are shown in Supplementary Table S1. The polymerase chain reaction (PCR) procedure was the same as that used in a previous study (Wang et al., 2017).

### Genetic Complementation and Artificial Suppression of *ZL16*

For the complementation of the *zl16* mutant, a full-length *ZL16* cDNA fragment including a 2.4-kb upstream sequence from the start codon of a genomic fragment were amplified with the HUQ and HUC prime pairs (Supplementary Table S2) using KOD FX polymerase (Toboyo, Osaka, Japan). Then the correct PCR products were cloned into the binary vector pCUbi1390 with restricted enzyme sites *Kpn*I/*BamH*I. The resultant recombinant construct pCUbi1390-*ZL16* was introduced into *Agrobacterium tumefaciens* EHA105. The *zl16* mutant *calli* was infected using the same method as described previously (Hiei et al., 1994).

A 20 bp fragment targeted to *ZL16* was designed using the CRISPR-P web tool^1^. The targeted fragment was inserted into the CRISPR/Cas9 vector pCAMBIA1305.1 carrying the CaMV35S promoter. The sequenced construct was introduced into cv. Nipponbare as described previously (Hiei et al., 1994). Positive T_1_ plant lines were obtained by sequencing the targeted gene.

### Heterologous Expression of Recombinant ZL16 Proteins in *E. coli*

The cDNA sequences from wild-type and mutant genes of ZL16 were amplified using the primer pairs pGEX-4T-2F and pGEX-4T-2R (Supplementary Table S2), respectively. The PCR products, containing each gene, missing the 56-amino-acid signal peptide, were inserted into the pGEX-4T-2 vector. The constructions pGEX:: ZL16 and pGEX:: zl16 were transformed into a competent *E. coli* BL21(DE3) strain (Genescript, Nanjing, China). Heterologous expression of the genes was induced at OD 0.6 with 1 mM isopropyl-beta-D-thiogalactopyranoside (IPTG). The cells were collected and lysed by sonication on ice. The protein was extracted from the resulting supernatant and purified by a glutathione S-transferase (GST) protein purification kit (Profinia^TM^, Bio-Rad, Hercules, CA, United States). HAD activities were assessed with a spectrophotometric method as described previously (Sharma et al., 2003; González-Thuillier et al., 2016). The substrate crotonoyl-CoA was purchased from Merck (Kenilworth, NJ, United States; catalog No. 28007). Protein concentration was determined by a BCA Protein Assay kit (Thermo Fisher Scientific, Waltham, MA, United States). SDS polyacrylamide gel electrophoresis (PAGE) was used to determine the purity of recombinant proteins.

### Subcellular Localization of ZL16 Protein

The online tools TargetP^2^ and ChloroP1.1^3^ were used to predict the transit peptide (Emanuelsson et al., 2007). To validate the subcellular localization of ZL16 protein, the coding sequence of *ZL16* (1-648, excluding the stop codon) was amplified using the primer pair *Zl16*- green fluorescent protein (GFP) (Supplementary Table S2). *ZL16* cDNA was inserted into the vector pET30a and then cloned into the transient expression vector pAN580 with the CaMV35S promoter to generate a chimeric gene at the N-terminus of the GFP as described previously (Hu et al., 2012). The constructs were then transformed to rice protoplasts as described previously (Yoo et al., 2007). Protoplast extraction was achieved using a method adopted in a previous study (Zhang et al., 2011). GFP fluorescence signals and autofluorescence of chlorophylls were analyzed with a confocal laser scanning microscope (LSM780, Carl Zeiss, Oberkochen, Germany). Empty vector pAN580 was used as the control.

### Quantitative Reverse Transcription-PCR (qRT-PCR) Analysis

QRT-PCR was conducted to investigate the expression of *ZL16*, and chlorophyll synthesis- and plastid development-related genes. Total RNA extraction from rice seedlings, cDNA preparation, and PCR analysis were performed as previously described (Zhou et al., 2012; Liu and Charng, 2013). The primers for the qRT-PCR are listed in Supplementary Table S3. The ubiquitin gene (*LOC_Os03g13170*) served as the internal control. The associated gene expressions were normalized to that of *ubiquitin*. For each sample, three technical replicates on three biological replicates were conducted.

### Phylogenetic Analysis

A ZL16 peptide sequence, with 216 amino acids, was obtained from Gramene^4^. A homologous analysis of ZL16 was conducted at the National Center for Biotechnology Information (NCBI, http://www.ncbi.nlm.nih.gov/).The peptide sequences were used to construct a phylogenetic tree using the MEGA version 5.0 software (http://www.megasoftware.net/; Peng et al., 2014) by the bootstrap method with 1,000 replicates.

### Modeling of the Three-Dimensional (3D) Structures

The 3D structures of zl16 and its wild type were modeled with the protein data bank (PDB) server^5^ and PDB KING viewer (Berman et al., 2000). The structural data of the *Plasmodium falciparum* HAD sequence (accession number UniProtKB Q965D7, Kostrewa et al., 2005; Maity et al., 2011) was used as a template for both rice sequences.

### RNA-Seq Library Preparation and Sequencing

Leaf samples from 8-day-old plant seedlings were ground immediately in liquid nitrogen and RNA was isolated using TriZol (Invitrogen) and cleaned with DNase1 using a Total RNA kit (Sigma-Aldrich). An Agilent 2100 BioAnalyzer was used to assess the quality of the resulting RNA. Library construction, PCR amplification and sequencing were conducted at Berry Hekang Biotech Ltd. (Beijing, China). Each RNA sample for sequencing library was repeated at least three times, and sequenced on the Illumina HiSeq^TM^ 2500 platform, which produced an average read length of 100 bp. The mapping of sequencing reads onto the rice genome (The MSU Rice Genome Annotation Project Data base version 7.0) was conducted using Bowtie software (bowtie2-2.2.9) with the default parameters (Langmead and Salzberg, 2012). A gene ontology (GO) functional classification for each sequence was determined using the Blast2go program (Conesa et al., 2005). Gene expression between the wild type and mutant was considered significantly different if the absolute value of | log_2_ (fold change) | was ≥1 and *q* ≤ 0.05.

### Measurement of the Fatty Acid Content

Fatty acids were extracted from rice seedlings and analyzed by the gas chromatography–mass spectrometry (GC-MS) of methyl ester derivatives as described previously (Bligh and Dyer, 1959). Lipids were extracted and separated by thin-layer chromatography (TLC). Each individual lipid was isolated and treated with methanolic HCl. Then the fatty acid methyl esters (FAMEs) were analyzed by GC-MS (TRACE^TM^ 1310, Thermo Fisher Scientific) with a 30-m (length) × 0.25-mm (inner diameter) × 0.25-μm (gel thickness) TG-5MS column. The parameters were set as following: sample size, 1 μL; injection temperature, 250°C; column temperature, 260°C; split ratio, 10:1; and carrier gas flow velocity, 0.5 mL/min. The fatty acids were detected by comparing retention times with FAME standards (Sigma, Aldrich, St Louis, MO, United States). Most tests were conducted at Qingdao Kechuang Quality Analysis Ltd. (Shandong, China).

### Statistical Analysis

Student’s *t*-tests were performed with Microsoft^TM^ Excel 2007. For each experiment, the number of biological replicates (*n*) is shown in the corresponding figure legend. *P* < 0.05 indicated a statistically significant difference and *P* < 0.01 indicated a very significant difference.

## Results

### Isolation and Phenotypic Description of the *zl16* Mutant

**FIGURE 1:**
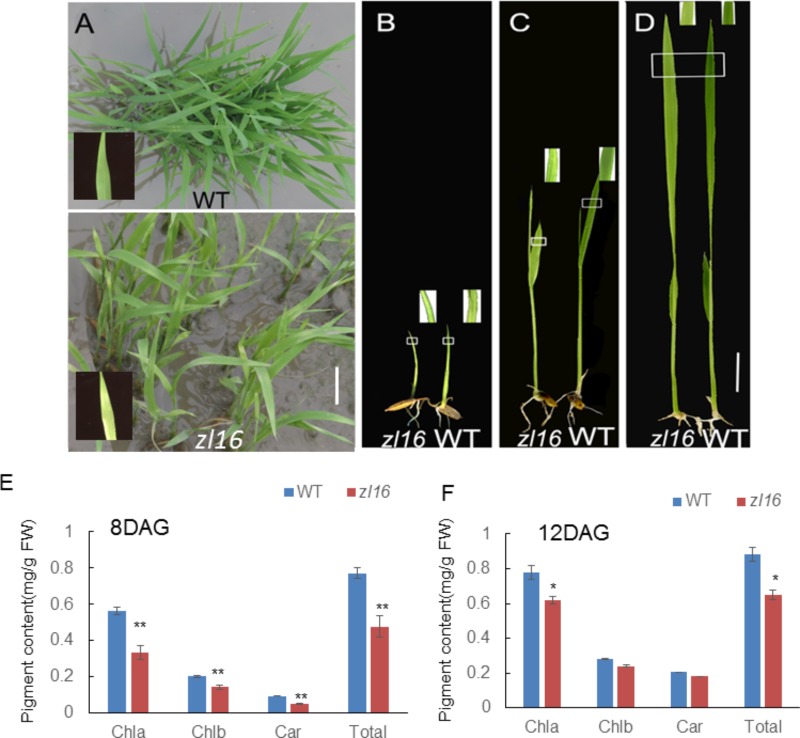
Phenotypic characteristics of *zl16* mutant. **(A)** Phenotypic comparison between the wild type (WT) and *zl16* mutant seedlings in the paddy field after 20 days of germination. The inset means enlarged detail of leaf blade. **(B–D)**The WT and *zl16* mutant seedlings in a growth chamber at the 5th- **(B)**, 8th **(C)**, and 12th-day after germination, respectively. Bars = 2 cm. The inset indicates enlargement of leaf sector in box. **(E–F)** Measurement of pigment contents in WT and *zl16* mutant seedlings at the 8th **(E)** and 12th- day **(F)** after germination (DAG), respectively. Chla, Chlorophyll a; Chlb, Chlorophyll b; Car, total carotenoids; FW, fresh weight. Bar denotes standard error (*SD*, *n* = 6). Asterisks indicate the significance levels according to Student’s *t*-test: ^∗^*P* < 0.05, ^∗∗^*P* < 0.01.

The *zl16* mutant was derived from the *indica* variety 93-11 mutagenized by EMS. Under high light intensity conditions (paddy field with a maximum light intensity of 3,000 μmol photons m^-2^ s), the *zl16* seedling displayed a transverse zebra leaf symptom during early growth (**Figure 1A**). As the leaf developed, the defective phenotype was gradually restored and disappeared after the fifth-leaf stage. Previously, a mutant (*zebra2*) was reported, which developed a zebra leaf phenotype under diurnal light–dark cycles in a natural field, whereas this was suppressed under shaded conditions (Chai et al., 2011; Han et al., 2012). Thus, *zl16* plants were grown in a growth chamber with an average of 30% of the light intensity of field conditions. Compared to those grown in a natural paddy field, the *zl16* seedlings exhibited much weaker lesions or stripes, and under growth chamber conditions, all seedlings displayed the light-green phenotype, with the most obvious symptoms observed at the second-leaf stage (**Figures 1B–D**). After the fifth-leaf stage, all leaves were as green as wild-type plants. When the plants initiated flowering, no leaf-color difference was found between the wild type and *zl16* mutant. Additionally, the mature *zl16* mutant plants showed a significant reduction in traits including flag and penultimate leaf areas, seed setting rate, and 1000 grain weight (**Table 1**), compared with the wild type (*t*-test, *n* = 10). Consistent with these observations, the *zl16* mutant leaves had a much lower chlorophyll and carotenoid contents than the wild-type seedlings at the earlier stages of leaf development (**Figure 1E**), but gradually recovered to wild-type levels in the later stages of development (**Figure 1F**).

**Table 1:** The agronomic trait comparison between the mature wild-type (WT) and *zl16* mutant plants.

Agronomic traits	WT	*zl16*
Flag leaf area (cm^2^)	85.1 ± 2.5	74.1 ± 4.3^∗∗^
Penultimate leaf area (cm^2^)	90.8 ± 8.15	85.4 ± 7.7^∗^
Seed setting rate (%)	92.4 ± 3.2	85.1 ± 0.4^∗∗^
1,000-grain weight (g)	32.9 ± 0.3	31.8 ± 0.2^∗∗^

*Data are means ± SD (*n* = 10). Student’s *t*-test was used for statistical analysis (^∗^*P* < 0.05; ^∗∗^*P* < 0.01)*.

To investigate the effect of different temperatures on the *zl16* phenotype, we treated *zl16* and wild type seedlings at two temperatures (i.e., high temperature, 30°C; low temperature, 20°C). We found that, despite much slower growth at the low temperature, the *zl16* mutant displayed a consistent leaf phenotype under different temperature conditions (Supplementary Figure S1). These results indicate that *zl16* is a temperature insensitive mutant.

### Chloroplast Ultrastructure of the *zl16* Mutant

**FIGURE 2:**
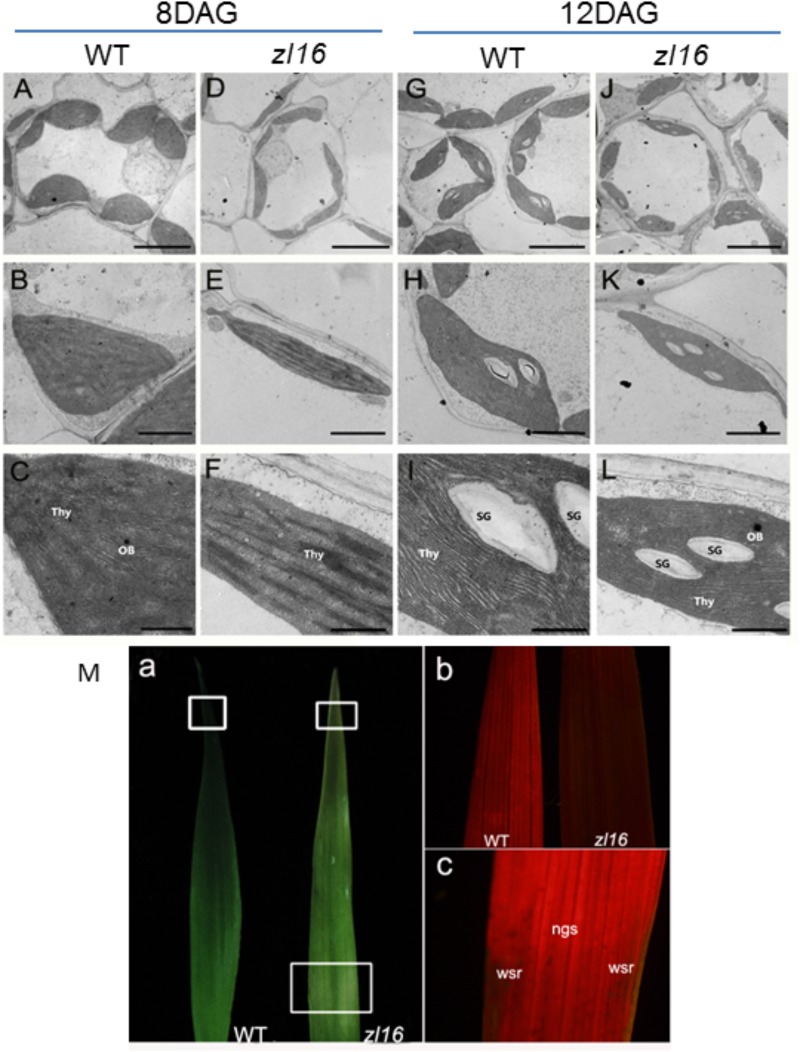
Ultrastructure of the chloroplast and chlorophyll autofluorescene observations of wild type (WT) and *zl16* mutant. **(A–L)** Transmission electron micrographs (TEM) images of WT (**A–C** and **G–I**) and *zl16* (**D–F** and **J–L**) chloroplasts. Seedlings were grown for 8 and 12 days after germination, respectively. OB, osmiphilic body; SG, starch granule; Thy, thylakoid lamellar. Bars: 2 μm **(A,D,G,J)**, 1 μm **(B,E,H,K)**, and 0.5 μm **(C,F,I,L)**. **(M)** Observation of autofluorescence of leaves. Panel **a**, observation of leaves under natural light conditions. The boxes in the upper and lower parts denote the regions that were enlarged in panel **b** and **c**, respectively. Panel **b**, autofluorescene of leaf tips from wild-type and *zl16* seedlings under UV light, respectively, at 8-d after germination. Panel **c**, autofluorescene from the white striped regions (wsr) and normal green sectors (ngs) in the *zl16* mutant seedlings under UV light, respectively.

To determine whether the color deficiency in the *zl16* mutant was related to ultrastructural changes in chloroplasts, we compared the chloroplast features from wild-type and *zl16* mutant leaves using TEM. As shown in **Figures 2A–C**, 8-day wild-type chloroplasts had well developed lamellar structures and were equipped with normal thylakoid membranes and stacked grana. In contrast, the chloroplasts in the white striped regions from 8-day mutant seedlings were obviously shriveled and lacked an organized lamellar structure (**Figures 2D–F**). As the seedlings developed, the chloroplast structural damage in the 12-day mutant leaves was alleviated to some extent and the chloroplast was almost restored to its wild-type status (**Figures 2G–I**).

A previous study showed that chlorophyll autofluorescence is an indicator of normal chloroplast development (Kitajima and Butler, 1975). We observed chlorophyll autofluorescence in wild type and *zl16* mutant seedlings under UV light (**Figure 2M** panels a–c). After 8 days of germination, chlorophyll autofluorescence at the leaf tip in the *zl16* mutant was obviously weaker than its counterpart in the wild type (**Figure 2M**, panel b). In the same way, the autofluorescence from the white striped regions was significantly lower than that in the normal green sectors of *zl16* leaves (**Figure 2M**, panel c). Collectively, the results indicated that chloroplast development was blocked in the *zl16* mutant.

### Isolation and Confirmation of the Rice *zl16* Gene

For a genetic analysis of the *zl16* locus, two F2 populations were produced from *zl16*/ Wuyunjing 7 and *zl16*/02428. All heterozygous F_1_ seedlings had the wild-type phenotype and the F_2_ segregation pattern fitted a 3:1 normal green to zebra leaf ratio under a *x*^2^ test (Supplementary Table S4). These data indicate that the zebra leaf in the *zl16* mutant is controlled by a single recessive nuclear gene.

**FIGURE 3:**
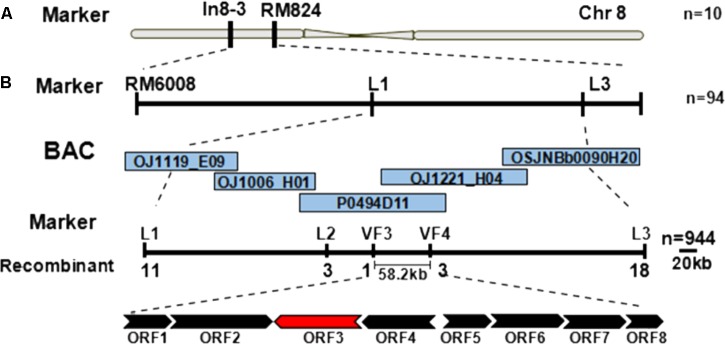
Map-based cloning of the *zl16* gene. **(A)** Rough mapping of the *zl16* locus with SSR and In-Del markers. *n*, the number of zebra leaf-like plants for mapping in the F_2_ population from *zl16*/02428 cross. Chr, Chromosome. **(B)** Fine-mapping of the *zl16* locus. BACs denote bacterial artificial chromosomes of rice whole genome. ORF, open reading frame; the most likely candidate gene is indicated in red. The arrows indicate transcriptional direction.

Genetic mapping of the *zl16* gene was conducted using the F_2_ population from the *zl16*/02428 cross. Initially the *zl16* locus was mapped to a region between the markers In8-3 and RM8243 on the short arm of chromosome 8 based on ten typical zebra leaf F_2_ individuals (**Figure 3A**). Then 94 additional zebra-leaf-like individuals were used to narrow the *zl16* lous into a 2.8 Mb distance between the markers L1 and L3. With the newly developed SSR and In-Del markers (Supplementary Table S1), as well as other 944 F_2_ homozygous mutant-like plants, we finally delimited the *zl16* locus to a 58.2 kb region between In-Del markers VF3 and VF4. Within this interval, eight open reading frames (ORFs) were predicted with the program RiceVarMap v2.0^6^ (**Figure 3B**, Supplementary Table S5).

**FIGURE 4:**
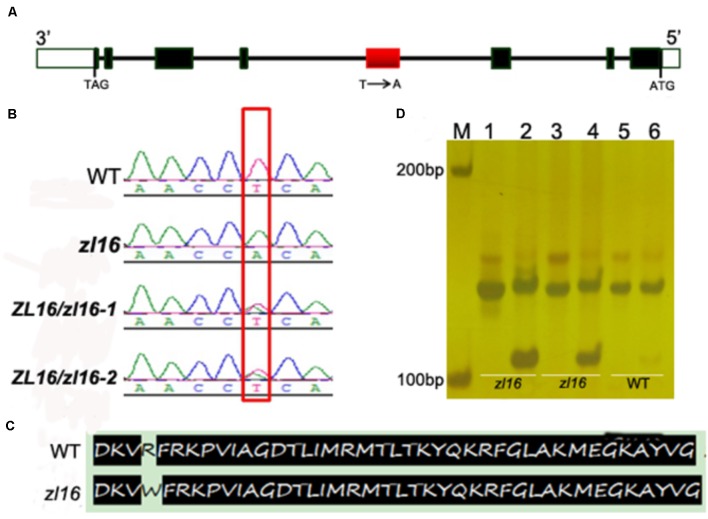
Identification of the mutation site and structure of *zl16.* **(A)** The structure of *ZL16* gene. ATG and TAG represent the start and stop codons, respectively. Black box indicates the exon and the line denotes intron. The region where base substitution happens is shown in red box. **(B)** cDNA sequence comparison between the wild type, homozygous *zl16* mutant (*zl16/ zl16*), and two heterozygous (*ZL16/ zl16*-1 and 2) genotypes. The point mutation was shown in box. **(C)** Comparison of translated protein sequences in the wild type (WT) and *zl16* mutant. **(D)** Identification of the base substitution between wild type and *zl16* with a dCAPs marker. Lane M means the molecular weight marker; Lanes 1, 3, and 5 indicate PCR products without *Alu*I digest; Lanes 2, 4, and 6, PCR products with *Alu*I digest. Lanes 1–4, DNA samples from the two homozygotes of the *zl16* mutant. Lanes 5–6, DNA samples from the wild-type cultivar, 9311.

To determine the mutation site, all eight ORFs were separately sequenced using genome DNA. Sequence alignments with wild-type genes revealed that an A-to-T transition in *LOC_Os08g12840* was present in the fourth exon of the third ORF in the *zl16* mutant (**Figures 4A,B**). This resulted in a substitution of Arg by Trp at the + 164 position of the encoding protein (**Figure 4C**). No difference was detected in the other seven ORFs.

To confirm the point mutation of *LOC_Os08g12840* in the *zl16* mutant, we designed a dCAPS marker and used it to genotype the wild type, heterozygous *zl16* plant, and homozygous *zl16* mutant. *Alu*I could cut the homozygous *zl16* allele but could not digest the wild-type allele (**Figure 4D**). We then used the dCAPS marker to detect zebra seedlings in the F_2_ population and found that all the recessive recombinants surrounding the *zl16* locus were co-segregated with this marker. Thus, *LOC_Os08g12840* (ZL16) was thought to be a candidate gene for the *zl16* phenotype.

**FIGURE 5:**
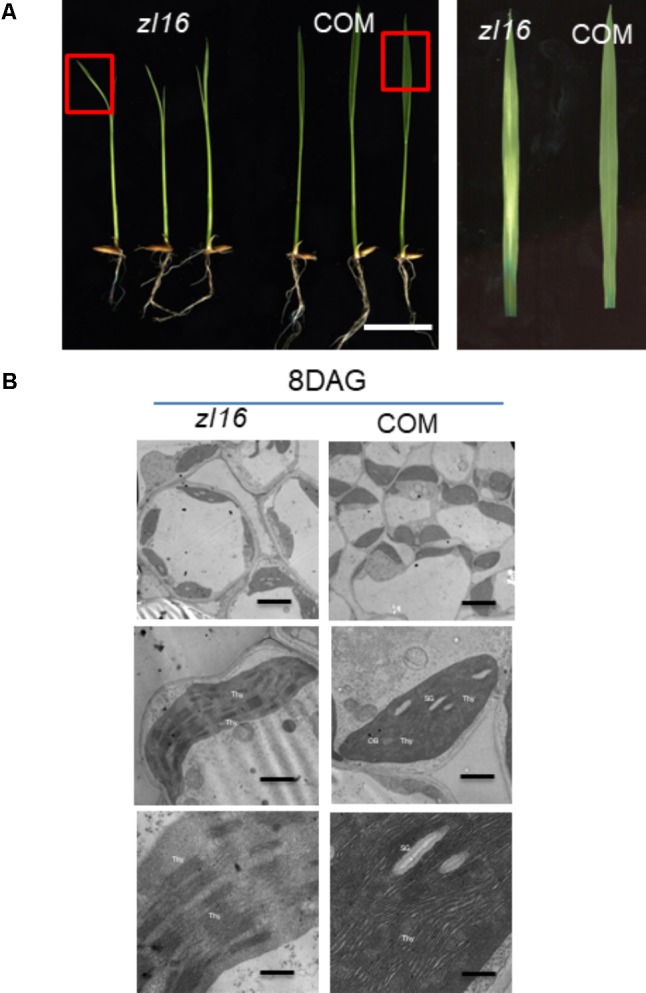
Functional confirmation of *ZL16* gene. **(A)** Genetic complementation. The functional wild-type (WT) ZL16 was inserted in the *zl16* mutant genome and *zl16* recover to normal green color. “COM” indicates three representative positive complemented plants generated by *callus* transformation. Bar = 2cm. The boxes in the left panel denote the regions that are enlarged in the right panel. **(B)**. Transmission electron micrographs (TEM) images of *zl16* (left panel) and complemented (COM) plant (right panel) chloroplasts. Seedlings were grown for 8 days after germination. OB, osmiphilic body; SG, starch granule; Thy, thylakoid lamellar. Bars: 2 μm (top row), 1 μm (middle row), and 0.5 μm (bottom row).

Complementation tests were conducted to examine whether the missense mutation led to the zebra phenotype. The seedlings regenerated from the *zl16* mutant *calli* that expressed the wild-type *ZL16* version restored the normal leaf color (**Figure 5A**) and chloroplast microstructure (**Figure 5B**), which was similar to the wild type. Homozygous lines of the transgenic plants with a CRISPR event were also isolated for analysis. Phenotyping results indicated that a knockouting of the *ZL16* gene by CRISPR-Cas9 demonstrated a chlorophyll deficient phenotype under a *japonica* rice ‘Nipponbare’ background (Supplementary Figure S2). Hence *LOC_Os08g12840* corresponds to the *ZL16* gene.

### Features of ZL16

**FIGURE 6:**
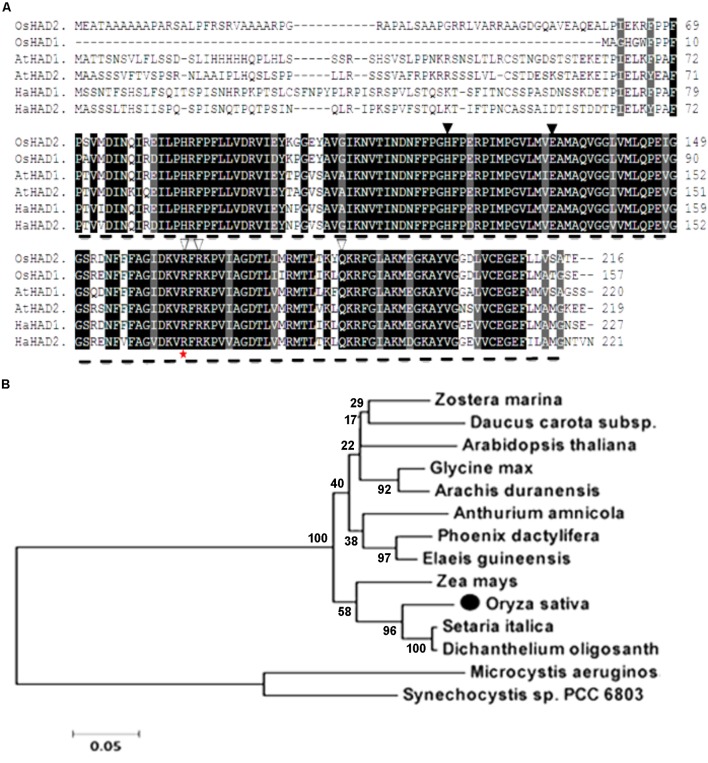
Alignment of the predicted amino acid sequence and phylogeny of ZL16. **(A)** Amino acid sequence alignment of ZL16 (O*sHAD1*). Dash line denotes FabA-like domain. Identical residues are highlighted as black shadings, highly conserved residues as dark gray shadings and weakly conserved residues as light gray shadings. Black triangles indicate the active sites, and white triangles are the residues participating in the ACP recognition and binding sites. The red star indicates the mutant site in the *zl16* mutant. Os, *Oryza sativa*; At, *Arabidopsis thaliana*; Ha, *Helianthus annuus* (sunflower). **(B)** Phylogenetic analysis of ZL16 proteins (black solid dot). A maximum likelihood phylogenetic tree was constructed based on the amino acid sequences using the MAGA5.0 software.

The whole amino acid sequence from ZL16 was used for a BLASTP search of the NCBI website^7^. We found that the *ZL16* gene had only a single copy in the rice genome, consisting of eight exons (**Figure 4A**) and encoding a polypeptide with 216 amino acid residues (**Figure 6A**). The ZL16 protein shared a high sequence similarity with its homologs in algae, monocotyledon, and dicotyledon (**Figure 6B**), suggesting its evolutionary sequence is highly conserved in plants. Many of these homologs were annotated as HAD; thus, we named ZL16 as OsHAD1 and another ZL16 homolog on chromosome 5, which was identified by BLAST analysis, as OsHAD2. OsHAD1 and OsHAD2 shared a 45.9% identity in amino acid sequences and little reference information is available for both OsHAD members in plants.

**FIGURE 7:**
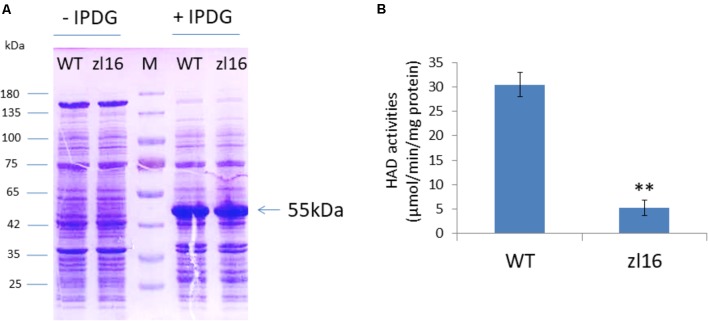
Heterologous expression of recombinant ZL16 proteins in *E. coli.* **(A)** Expression of wild-type (WT) and zl16 recombinant proteins in *E. coli*. –/+ denote before and after adding 1 mM isopropyl-beta-D-thiogalactopyranoside (IPTG) for protein induction. M means molecular weight marker. Arrow indicates the target protein from ZL16 or zl16 (29 kDa) fusing with glutathione S-transferase (26 kDa). **(B)** Comparison of β-hydroxyacyl-ACP dehydratase (HAD) enzyme activities between the wild type (WT) and zl16 after purification of the recombinant proteins. Data are means ± SD of six replicates. ^∗∗^*P* < 0.01.

ZL16 contains a FabA-like domain and belongs to the hot-dog protein superfamily. There are two critical areas in the FabA-like domain. One is considered to be a catalytic activity center and includes two conserved residues (His119 and Glu133), while the other is considered to be involved in ACP recognition and binding, and harbors three critical residues (Arg164, Arg166, and Glu181) (González-Thuillier et al., 2016). The point mutation in the *zl16* mutant (Arg164Trp) was found to reside at the first conserved residue in the ACP recognition and binding sites (**Figure 6A**). Based on the 3D structure model, the mutation in *zl16* led to the enlargement of a curve angle near the + 164 site in the tertiary structure compared to the wild type (Supplementary Figure S3), which might compromise HAD recognition and its binding ability to its substrates. To further verify the hypothesis, we successfully expressed the wild-type and mutant *zl16* coding proteins in *E. coli* with the expected molecular weight sizes (**Figure 7A**). A comparison of the enzyme activity experiments revealed that purified mutant *zl16* recombinant protein had a significantly lower activity than its wild-type counterpart, which amounted to only 17.1% of the wild-type level (**Figure 7B**). The near loss of HAD enzyme activity in the mutant was consistent with the recessive genetic feature of the *zl16* mutation.

**FIGURE 8:**
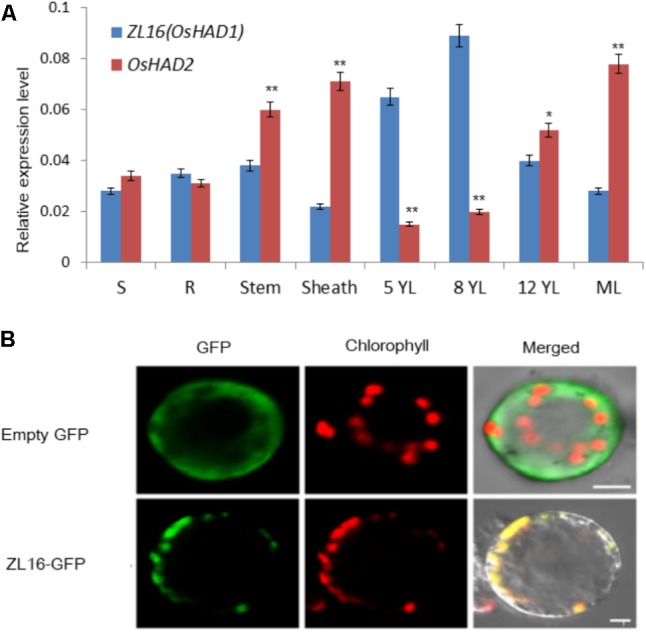
Expression pattern and subcellular localization of ZL16. **(A)** Expression analysis of *ZL16*(*OsHAD1*) and *OsHAD2* genes in rice various tissues. S, seed; R, young root; ML, mature leaf during flowering; 5, 8 and 12 YL, young top leaves from 5-, 8- and 12-day-old seedlings after germination. *Ubiquitin* gene was used as an internal control. Data are means ± SD of three biological replicates. For *t*-test, *OsHAD2* values were compared with expression level of *ZL16*(*OsHAD1*). ^∗^*P* < 0.05; ^∗∗^*P* < 0.01. **(B)** Transient expression of ZL16–GFP in rice protoplasts. Empty GFP indicates vector construct from GFP alone for subcellular localization, ZL16-GFP indicates construct from fused ZL16 and GFP for subcellular localization. GFP, GFP signal of ZL16 fusions; Chlorophyll, chlorophyll autofluorescence; Merged, merged images of GFP and chlorophyll autofluorescence. Bar = 10 um

QRT-PCR revealed that *ZL16* transcripts were detected in all tissues examined including seed, root, sheath, stem, mature leaf, and young leaf, with a relatively preferential expression in the young leaf (**Figure 8A**). During seedling growth, *OsHAD1* reached the highest expression level in 8-day-old seedlings, and then decreased as the plant developed. In contrast, *OsHAD2* showed low expression during early seedling growth, but there was a constant increase during leaf development. *OsHAD2* had a higher or equal expression than *OsHAD1* in all tissues other than young leaves.

Both online tools, ChloroP and TargetP, predicted ZL16 harbors a chloroplast transit peptide at its N-terminus, suggesting ZL16 is localized to chloroplasts. To verify this, we generated the full-length *ZL16* cDNA (not including the stop codon) fusion with GFP. In rice protoplasts, confocal microscopy showed that the green fluorescence of GFP-ZL16 was entirely co-localized with the autofluorescence signal of chlorophyll in the chloroplasts, while the empty GFP protein was mainly located in the cytosol (**Figure 8B**). These results indicate that ZL16 is located within the chloroplast, which is a cellular compartment where fatty acids are mainly synthesized in plants (Ohlrogge and Jaworski, 1997).

### Expression Analysis of Other Related Transcripts

**FIGURE 9:**
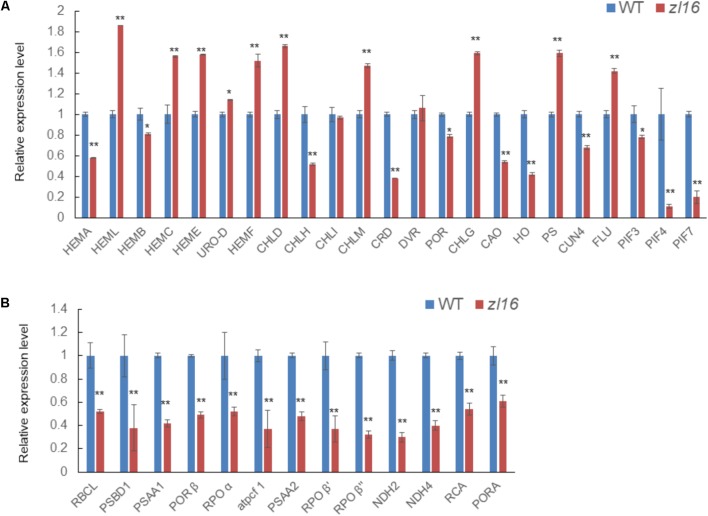
Expression analysis of genes associated with chlorophyll biosynthesis and plastid development in the wild type (WT) and *zl16.* **(A)** Relative expression of genes involved in chlorophyll biosynthesis. **(B)** Relative expression of genes involved in plastid development. Experiments were normalized using the *Ubiquitin* gene as an internal control. Data are means ± SD of three replicates.

The *zl16* mutant had a zebra leaf lesion and produced less chlorophyll than its wild type (**Figure 1**), and therefore it was presumed that the mutant might have transcriptional defects in chlorophyll synthesis. We performed a qRT-PCR to analyze the expression of chlorophyll synthesis-related genes. In 23 tested genes, 11 were down-regulated: HEMA (encodes glutamyl-tRNA reductase), HEMB (encodes porphobilinogen synthase), CHLH (encodes Mg-chelatase subunit H), CRD [encodes Mg-protoporphyrin IX monomethyl ester (oxidative) cyclase], PORA (encodes NADPH:protochlorophyllide oxidoreductase A), CAO (encodes chlorophyll a oxygenase 1), HO (encodes heme oxidase 1), GUN4 (encodes GENOMES UNCOUPLED 4), PIF3, PIF4, and PIF7 (encode phytochrome-interacting factors 3, 4, and 7, respectively). Of these genes, PORA was the most down-regulated (**Figure 9A**). These results indicate that chlorophyll biosynthesis might be impaired in the *zl16* mutant.

In the TEM observation, chloroplast development of the mutant was found to be abnormal relative to the wild type (**Figure 2**). Previous studies have revealed that impaired plastid development could trigger plastid retrograde signaling to regulate nuclear-encoded gene expression (Chi et al., 2013), and we speculated that the transcription levels of some relevant genes in the mutant might be altered. As expected, the qRT-PCR revealed that 13 detected genes participating in plastid development were all significantly down-regulated in 8-day-old *zl16* seedlings (**Figure 9B**), suggesting an important role for *ZL16* in the development of rice plastids.

To obtain a comprehensive view of the transcriptome affected by the *zl16* mutation, we conducted an RNA-Seq analysis to detect the differential genes between the *zl16* mutant and its wild type. Under stringent conditions (*q* < 0.001; |log_2_ (fold change)| > 3), a total of 195 differential genes encoded by the nuclear genome were identified, among which 124 were up-regulated and 71 down-regulated. The GO analysis of these down-regulated genes revealed that, apart from chlorophyll biosynthesis and plastid development, most of them were involved in the biosynthesis of cell constituents, especially for membrane systems (Supplementary Figure S4).

### Determination of the Fatty Acid Content

**FIGURE 10:**
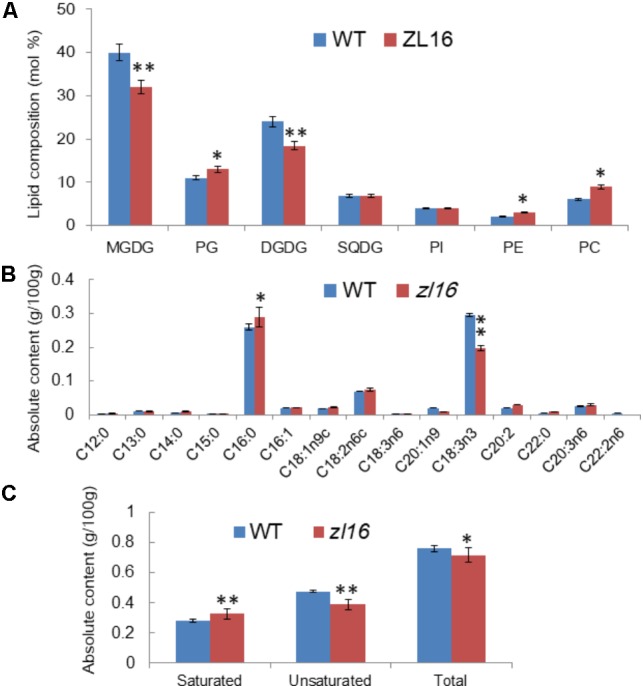
Determination of fatty acids in wild-type (WT) and *zl16* seedlings. **(A)** Comparison of polar lipid classes (percentage of total polar glycerolipids analyzed) in leaves of the wild type and *zl16* mutant. The bars indicate means ± SD of three measurements. MGDG, monogalactosyl-diacylglycerol; DGDG, digalactosyl-diacylglycerol; PC, phosphatidylcholine; PE, phosphatidylethanolamine; PG, phosphatidylglycerol; PI, phosphatidylinositol; SQDG, sulfoquinovosyldiacylglycerol. **(B)**The content of each fatty acid in wild-type and *zl16* mutant seedlings. **(C)** Comparison of saturated and unsaturated fatty acids in wild-type and *zl16* mutant seedlings. *n* = 6. Asterisks indicate the significance levels according to Student’s *t*-test: ^∗^*P* < 0.05, ^∗∗^*P* < 0.01.

To further investigate the function of *ZL16*, we measured the kinds and contents of various fatty acids in wild type and mutant seedlings at 8 days after germination. TLC analysis revealed in the mutant the contents of galactolipids [monogalactosyl-diacylglycerol (MGDG) and digalactosyl-diacylglycerol (DGDG)] were reduced by 25 and 21%, respectively, compared to wild type. In contrast, a slight increase (ranging from 6∼10%) in the abundance of other membrane lipids such as phosphatidylcholine (PC), phosphatidylglycerol (PG), and phosphatidylethanolamine (PE) were observed, while the levels of sulfoquinovosyldiacylglycerol (SQDG) and phosphatidylinositol (PI) were not changed significantly in the mutant (**Figure 10A**). Because the two galactolipids (MGDG and DGDG) and SQDG are main components of thylakoid membrane lipids (Kobayashi, 2016); this result suggests that the mutant had a disturbed chloroplast membrane lipid composition relative to wild type plants.

A total of 13 different fatty acids were identified in the wild type seedlings, among which four (C13: 0, C14: 0, C16: 0, and C22: 0) were saturated fatty acids, and the remainder were unsaturated fatty acids. In *zl16* seedlings, a total of 14 different fatty acids were identified. Compared with the wild type, two more saturated fatty acids (C12: 0 and C15: 0) were present, but an unsaturated fatty acid (C22: 2n6) was missing in the mutant. Remarkably, in contrast to the wild type where the most abundant fatty acids was octadecenoic acid (C18: 3n3, an unsaturated fatty acid), palmitic acid (C16: 0, a saturated fatty acid) was predominant in the mutant (**Figure 10B**). A previous study showed that C18: 3n3 is a major component of MGDG in the chloroplast membrane (Xu et al., 2010). Thus, the significant reduction of C18: 3n3 in the mutant was consistent with the impaired chloroplasts. Overall, the total fatty acid content of the mutant was reduced relative to the wild type. Moreover, we found that the saturated fatty acid content was significantly higher and the unsaturated fatty acid was significantly lower than in the wild type (**Figure 10C**).

## Discussion

Many chlorophyll- and chloroplast-related mutations that change leaf coloration and/or seedling viability have been identified and designated as *virescent*, *stripe*, *albino*, *chlorina*, *zebra*, and *yellow variegated* based on visual phenotypes. Among them, some (such as *virescent1*, *2*, and *3*) have been reported as temperature-conditional (Iba et al., 1991; Kusumi et al., 1997; Sugimoto et al., 2004), while others (such as LCM6, 7, and 8) are thermo-insensitive (Wu et al., 1999) in terms of their different responses to temperature. Zebra mutants have alternating green and white (or yellow) stripes on leaf blades and had been reported in many monocotyledonous crops, including rice, maize, sorghum, and pearl millet (Coe et al., 1987; Werner and Burton, 1991; Oki et al., 1997; He et al., 2000; Kusumi et al., 2000). At least 15 non-allelic “zebra” mutants (*zebra*1-15) have been identified in rice, and the causative genes had been mapped on different chromosomes^8^. Two of them, *ZEBRA-NECROSIS* and *ZEBRA2*, which encode a thylakoid-bound protein (Li et al., 2010) and carotenoid isomerase (Chai et al., 2011; Han et al., 2012), respectively, were cloned. In addition to temperature, the appearance of the zebra phenotype also depends on light and the seedling growth stage (He et al., 2000; Kusumi et al., 2000). In this study, the rice *zebra leaf* mutant *zl16* was identified. Under field conditions with high light intensity, *zl16* exhibited its characteristic zebra phenotype with reduced chlorophyll content, while mutant plants grown in a growth chamber under a low light intensity displayed a much less severe phenotype (**Figure 1**). Moreover, we found that the temperature treatment did not enhance the symptom in the *zl16* mutant (Supplementary Figure S1). These results indicate that the *zl16* phenotype is light-dependent rather than temperature-conditional.

Map-based cloning indicated that *ZL16* encodes a HAD, which is one of four enzymes (KAS, KAR, HAD, and ENR) in the carbon chain elongation cycle of *de novo* fatty acid synthesis. Fatty acids and their derivatives can not only function as an important component of membrane lipids that are vital for cell growth and development, but also play an essential role as signaling molecules (Weber, 2002; Kachroo et al., 2003, 2004). In *Arabidopsis*, several mutants have been reported in the different steps of the fatty acid synthesis. Downregulation of *KAS* expression in a *kas1* mutant due to a T-DNA insertion in the 5′-UTR of *KAS1* causes chlorotic and curly leaves, decreased fertility, and significantly suppressed chloroplast division in rosette leaves (Wu and Xue, 2010). A *mod1* mutant, with a point mutation in the sixth exon of *ENR*, produces reduced ENR activity, variegated leaves, abnormal chloroplast structure, and reduced fertility (Mou et al., 2000). Under UV light, the mesophyll cells in *mod1* yellowish leaves irradiated a weak red fluorescence (Mou et al., 2000). Similarly, here we found a single nucleotide substitution in a conservative region of HAD that led to the reduction of both enzyme activities and total fatty acids, altered the expression of photosynthesis-related genes, and resulted in a defective development of the chloroplast during early leaf development in rice. Intriguingly, apart from leaf color, in the *zl16* mutant there was also a significant reduction in traits such as the size of mature flag and penultimate leaves, seed setting rate, and 1,000-grain weight (**Table 1**), which are highly consistent with the *Arabidopsis mod1* mutant (Mou et al., 2000). These results indicate that a lipid supply is essential to plant growth and development including the chloroplast structure in plants. There is an abundance of thylakoid membranes in chloroplasts; thus, sufficient provision of lipids is required for normal chloroplast formation. Defects in the synthesis of fatty acids can compromise chloroplast membrane assembly and development (Fan et al., 2015), in accordance with the observation of significantly reduced MGDG and DGDG in this study. It is worth noting that MGDG and DGDG account for about 50 and 25% of total thylakoid lipids, respectively, in plants (Kobayashi, 2016). The lack of the two galactolipids in the *zl16* mutant confirms the close relationship between polar lipid supply and chloroplast development. Additionally, our RNA-Seq results indicated that many genes associated with the biosynthesis of cell membrane systems were downregulated in the *zl16* mutant, which further supports the notion that a lipid supply is essential for ensuring chloroplast structure.

Chloroplast development was blocked in *zl16* leaves at early growth stages; however, it recovered to some extent in terms of chloroplast size in the later developmental stages relative to the wild type. This is coincident with leaf color phenotypic changes in the *zl16* mutant. There are two possible reasons for the restoration of chloroplasts. First, in the course of ZL16 (OsHAD1) deficiency, another OsHAD1 paralogous member (OsHAD2) might replenish the HAD activity in plastids. We found that ZL16 is predominantly expressed in young leaves, whereas OsHAD2 was mainly present in mature leaves based on qRT-PCR data, indicating that ZL16 have a function in fatty acid synthesis primarily in the early stages, while OsHAD2 is more important in the later stages of plant development. Another explanation for the presence of normal chloroplasts is that the mitochondrial fatty acid synthesis system may compensate for the defective plastidial fatty acid synthesis. In a plant cell, *de novo* fatty acid synthesis can occur in both plastids and mitochondria (James et al., 1995). Similarly to plastids, mitochondria contain all the enzymes associated with *de novo* fatty acid synthesis and can function independently to form long-chain acyl-ACP (Wada et al., 1997; Gueguen et al., 2000; Focke et al., 2003). The central function of mitochondrial fatty acid synthesis is to maintain lipoic homeostasis, which is important for supporting photorespiration (Ewald et al., 2007; Guan et al., 2015; Guan and Nikolau, 2016). Recently Guan et al. (2017) showed that in both *Arabidopsis mthad* and *mtkas* mutants, the thylakoid membrane assembly is altered relative to their wild types, and is accompanied by the appearance of many small starch granules in the chloroplasts. The deformed chloroplasts are similar to those in the *zl16* mutant here. Given the functional conservation of HAD for chloroplast development between dicot and monocot plants, we speculate that in the absence of rice plastidial HAD activity (i.e., OsHAD1/ZL16), mitochondrial FA synthesis, e.g., from OsHAD2, may partially complement this gap in fatty acid chain elongation.

In summary, this study found that *ZL16*, encoding a *HAD* in rice, plays a vital role in the synthesis of fatty acids and sets a foundation for connecting the relations between lipid and chloroplast development in rice leaves. This information will be useful for gene engineering to improve photosynthetic ability in rice. Further studies should be conducted to obtain comprehensive lipidomic data, particularly focusing on chloroplast lipids, and so enable a mechanistic understanding of the findings.

## Author Contributions

LL, JW, and ZL conceived the research. JW supervised the whole project. ZL, LL, ZW, HG, JY, MH, YZ, ZZ, SL, LC, XL, and YT performed the experiments. JW and YW provided the mutant materials. YW, SL, LC, XL, YT, SZ, and JL involve technical assistance. ZL analyzed the data and drafted the manuscript. LL analyzed the data and rewrote the manuscript completely.

## Conflict of Interest Statement

The authors declare that the research was conducted in the absence of any commercial or financial relationships that could be construed as a potential conflict of interest.

## Footnotes

1 http://crispr.hzau.edu.cn/CRISPR2/

2 http://www.cbs.dtu.dk/services/TargetP/

3 http://www.cbs.dtu.dk/services/ChloroP/

4 http://www.gramene.org/

5 http://www.rcsb.org/pdb/

6 http://ricevarmap.ncpgr.cn/cgi-bin/gb2/gbrowse/ricevarmap2/

7 https://www.ncbi.nlm.nih.gov/

8 http://www.shigen.nig.ac.jp/rice/oryzabase/

## Abbreviations

ACP: acyl carrier protein
DAF: days after flowering
DAG: days after germination
ENR: enoyl-ACP reductase
FAS: fatty acid synthase
HAD: β-Hydroxyacyl-ACP dehydratase.
