# Supplementary material for: Identification and Phenotypic Characterization of ZEBRA LEAF16 Encoding a β-Hydroxyacyl-ACP Dehydratase in Rice
Source: Front Plant Sci. 2018 Jun 12;9:782. doi: 10.3389/fpls.2018.00782 (PMC6005893; doi:10.3389/fpls.2018.00782)
Supplement: Supplementary file 1 [file Data_Sheet_1.docx]

Supplementary Material

*ZEBRA LEAF16,* encoding a β-hydroxycyl-ACP dehydratase, is involved in rice chloroplast development

Ziwen Liu^1^, Zhiyuan Wang^1^, Han Gu^1^, Jia You^1^, Manman Hu^1^, Yujun Zhang^1^, Yihua Wang^1^, Shijia Liu^1^, Liangming Chen^1^, Xi Liu^1^, Yunlu Tian^1^, Ling Jiang^1^ and Jianmin Wan^1,2^ ,Linglong Liu^1,*^

*** Correspondence:** Linglong Liu, [liulinglong@njau.edu.cn](mailto:liulinglong@njau.edu.cn)

# Supplementary Figures and Tables

## Supplementary Figures

##
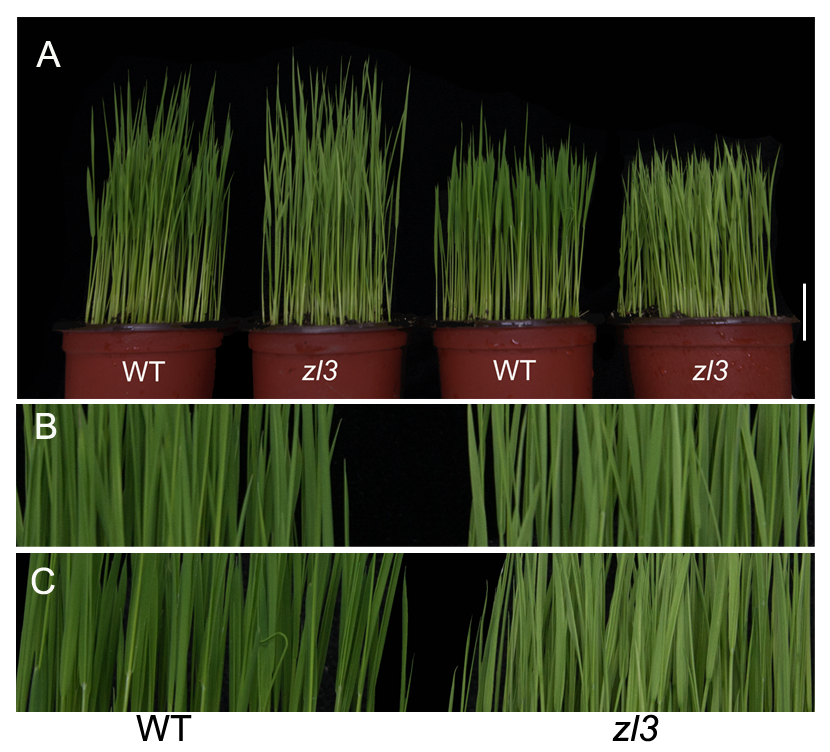


*zl16*

*zl16*

WT

WT

30 ℃

20 ℃

**Supplementary Figure S1.** The seedlings of the wild type (WT) and *zebra leaf 16* (*zl16*) mutant under different growth temperatures. Bars= 5 cm.

**
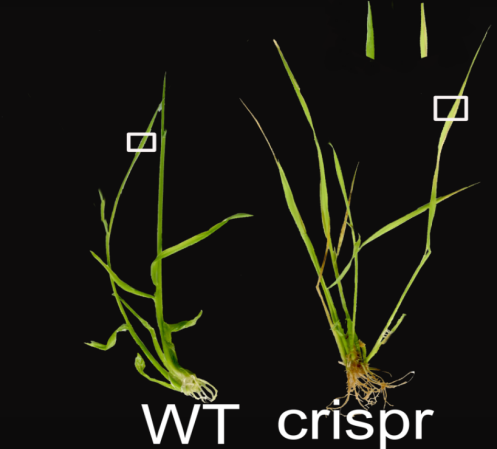
**

**Supplementary Fig. S2.** Knock-out of the *ZL16* gene by a CRISPR/Cas9 system. The line *crispr* is a representative homozygous mutant in cv. Nipponbare background produced by CRISPR/Cas9. The inset indicates enlargement of leaf sector in box.


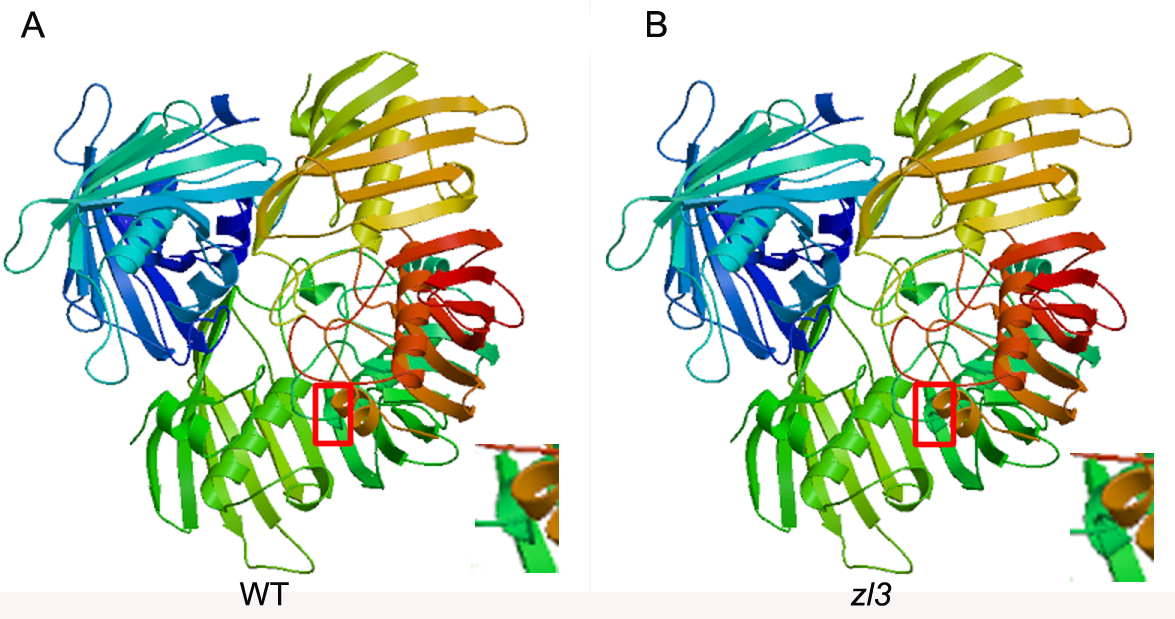


WT

*zl16*

**Supplementary Fig. S3.** Predicted three-dimensional structure of *β*-hydroxyacyl-ACP dehydratase (HAD) in WT (A) and *zebra leaf 16* (*zl16*) mutant (B)*.* The red rectangle represents structure difference between the wild type and the mutant.


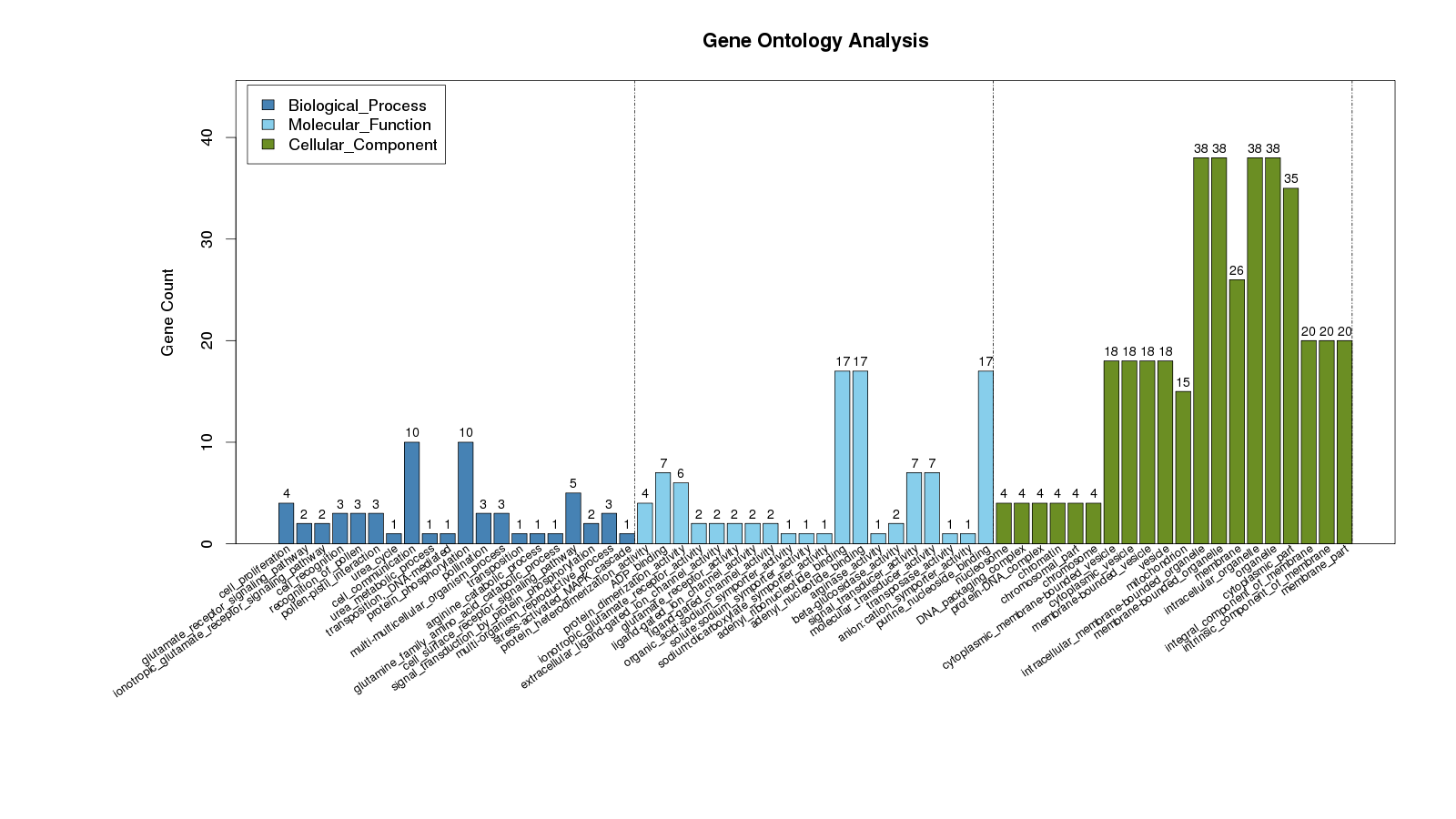
 **Supplementary Fig. S4.** [Gene ontology](http://web.mit.edu/~r/current/arch/i386_linux26/lib/R/library/limma/html/goana.html) (GO) analysis of down-regulated differential genes between the wild type and *zl16* mutant.

## Supplementary Tables

**Supplementary Table 1** Primers sequences for fine mapping

| Marker | Forward sequence (5’-3’) | Reverse sequence (5’-3’) |
| --- | --- | --- |
| In8-3 | TTTTAAAGCTGCGCCAAAAG | CATAACCGGTAAAGGAGTAGCC |
| RM8243 | CTCGTGCAACCATTATATTC | ACCTTAGCTGTCCTGAATTG |
| RM6008 | AGAGAAGAGAGAAGCGCACG | CATACATCACACGAGGACGG |
| L1 | CTTTTTAGGCAAATGACTCT | TTCTCTACACGCACAAACTA |
| L2 | TAAAAAGATTGACTGGAAGG | TGCTGAGTACCTTGCTAAA |
| L3 | GAGAATGAGCGATGATGAGA | AAAATGTCTGAAAATGTGGC |
| VF3 | GAAAGCAGAAGCACCACCAA | TGGATTGAGACCAGCATGAGT |
| VF4 | TGTGTACAACCTTTGTGGGT | TGTGCCTTTGATACCAACCA |
| dCAPS | ATCCCCTGCAATTACTGGCTTACGGAAGC | ACCTTTATGCACAGGCTATGGCCCAGGTT |

| **Supplementary Table 2** Primers for vector construction | | |
| --- | --- | --- |
| Vector | Primer name | Prime sequence(5’-3’) |
| pCUbi1390 | HuQ：F | CCGGCGCGCCAAGCTTCTTTGGGGAGGGTGGTGTCAGG |
|  | HuC：R | TGGTCCAACCTCCTGAGCCTCCACCGCCTG |
|  | HuBu-cDNA：F | TCAGGAGGTTGGACCAGCCTCGTTCCTATCG |
|  | HuBu-cDNA：R | GAATTCCCGGGGATCCTTACAACAGCAACCACAGCCAGA |
| pCAMBIA1305.1 | CrispR-*Zl16*: F | AGATGATCCGTGGCACCCTCCCTTCCCGAGCGTCAGTTTTAGAGCTATGC |
|  | CrispR-*Zl16*: R | GCATAGCTCTAAAACTGACGCTCGGGAAGGGAGGGTGCCACGGATCATCT |
| PAN580GFP | *Zl16*-GFP：F | CGGAGCTAGCTCTAGAGAAGCCAAACCCACAAAACCCC |
|  | *Zl16*-GFP：R | TGCTCACCATGGATCCTTCGGTTGCGCTAACCAGGAGG |
|  | GFP-*Zl16*：F | CGAGCTGTACAGATCTTTGGACCAGCCTCGTTCCTATCG |
|  | GFP-*Zl16*：R | GCCAAATGTTTGAACTGCAGCTATTCGGTTGCGCTAACCAGGA |
| pGEX-4T-2 | pGEX-4T-2：F | GGTTCCGCGTGGATCCATGGAGGCCACCGCCGCCGC |
|  | pGEX-4T-2：R | GTCGACCCGGGAATTCCTATTCGGTTGCGCTAACCAGG |

| **Supplementary Table 3** Primers for quantitative RT-PCR | | | |  |  |
| --- | --- | --- | --- | --- | --- |
| Gene name | Abbr. | Forward sequence (5’-3’) | Reverse sequence (5’-3’) | | GenBank No.  (NCBI) |
| *ZEBRA LEAF 16* | *ZL16* | ACCAGCCTCGTTCCTATCGCC | TTACAACAGCAACCACAGCCAGA | | Os08g0225000 |
| *OsHAD2* | *OsHAD2* | TGTGATTGCTGGAGACACCT | CAGATCCGGTTGCCATCAAG | | [Os05g0435700](http://rapdb.dna.affrc.go.jp/viewer/gbrowse_details/irgsp1?name=Os05g0435700) |
| *Glutamyl-tRNA reductase* | *HEMA* | GATGCAATCACTGCTGGAAAGCGT | CCATCTTGCCAGCACCAATCAACA | | Os10g0502400 |
| *Porphobilinogen synthase* | *HEMB* | TGGCATTGTCAGGGAAGATGGAGT | CCAAAGCAGCACGTATTGCTCCAA | | Os06g0704600 |
| *Hydroxymethylbilane synthase* | *HEMC* | TCATTCCGAGGGCTATTGGCTTCA | ACACTCTAGTTGGCCAATGGTGGA | | Os02g0168800 |
| *Uroporphyrinogen III decarboxylase* | *HEME* | AATGGAGGCTTGCTTGAGCGAATG | TTGTTACCAAGGCGTCTCCTTCCA | | Os01g0622300 |
| *Coproporphyrinogen III oxidase* | *HEMF* | ACTGACTGCACGATGGCAGTATGA | AGAGATCGAGCCATTCCTTTGGGT | | Os04g0610800 |
| *Glutamate-1-semialdehyde-2,1-aminomutase* | *HEML* | AGAACAAAGGGCAGATTGCTGCTG | TGTTTCGTCAAGTCACGGAGAGCA | | Os08g0532200 |
| *Uroporphyrinogen decarboxylase* | *URO-D* | AGGCTTCCACTGACAGGTGTTGAT | AAAGAACGCCAGGGTCAACATTCC | | Os03g0337600 |
| *Mg-chelatase subunit D* | *CHLD* | TAGCACAGCTGTCAGAGTGGGTTT | TTGCCAGCCACCTCAAGTATCTCA | | Os03g0811100 |
| *Mg-chelatase subunit G* | *CHLG* | CCAGCCACTGATGAAAGCAGCAAT | AGAGCGCTAATACACTCGCGAACA | | Os05g0349700 |
| *Mg-chelatase subunit H* | *CHLH* | GCACGGGAACTTGGCGTTTCATTA | ACATGTCCTGGAGCTGCTTCTCAT | | Os03g0323200 |
| *Mg-chelatase subunit I* | *CHLI* | AGGGATGCTGAACTCAGGGTGAAA | AAGTAGGACTCACGGAACGCCTTT | | Os03g0563300 |
| *Mg -protoporphyrin IX methyltransferase* | *CHLM* | GCTTCATCTCCACGCAGTTCTACT | GCAATGACGAATCGAAGACGCACA | | Os06g0132400 |
| *Mg-protoporphyrin IX monomethyl ester (oxidative) cyclase* | *CRD* | TGGATCTAACATGACACGCACCCA | ACTGTAACGGCATTCTTCTCCGGT | | Os01g0279100 |
| *Divinyl Reductase* | *DVR* | TTCTTCGAGAGGGTGATCAGGGAA | GAAACTGGCAATGGCAGCCAAGAA | | Os03g0351200 |
| *Protochlorophyllide oxidoreductase A* | *PORA* | TCGTCGGCCTCGTCTGAGTTTATT | AGGCCTCTCTCACTGAAAGCTGAA | | Os04g0678700 |
| *Chlorophyll a oxygenase* | *CAO* | ACACCTTCATCTGGGCTTCAAGGA | AGATGCGTCGAACATTGCTTGGTG | | Os10g0567400 |
| *Heme oxygenase 1* | *HO* | TGGAGGCCGAATGATTGGGAAGAA | AGGCGACTTCGTTAAGCTTGTTGC | | Os06g0603000 |
| *Photoperiod-sensitivity-13* | *PS* | TTCCCTGAATACGCCCGTGATGAT | AGATTGATCAGGCCGAAAGGC | | Os01g0949400 |
| *Fluorescent1* | *FLU1* | TGTTACAGAAGCCTACGGTGCGAT | CTCAAAGGTTGCACACACGGTTCT | | Os01g0510600 |
| *Genomes uncoupled 4* | *GUN4* | AAGGGAAGGAGAGGCCAAAGTTCA | ACCATGACCAGCATCTCTGCATCA | | Os11g0267000 |
| *Phytochrome interacting factor 3* | *PIF3* | TGGGCAAACAATTCCTTCGTCAGC | CTTGTTGCTGGTCTTGCACCATGT | | Os01g0286100 |
| *Phytochrome interacting factor 4* | *PIF4* | TGATCACCAGGAAGCAGAGGCTTT | TGGTGTTCCAATCAGGGACGAACT | | Os12g0610200 |
| *Phytochrome-interacting factor7* | *PIF7* | AGCAATCCCTTCCTTCACCCAGAT | AGTGCTAGCTGACGCTTTCGGTAT | | Os03g0782500 |
| *Photosystem I P700 chlorophyll a apoprotein A1* | *PsAA1* | GGGAGGTGGCGAGTTAGTAG | AATGCGTGAATGTGATGGAC | | OsP1g00340 |
| *Photosystem I P700 chlorophyll a apoprotein A2* | *PsAA2* | TTATCTTCAACGAGCGGT | TATCTCCAGGTCCTATTGTT | | OsP1g00330 |
| *Photosystem II D2 protein* | *PsBD1* | CTGCTACTGCTGTTTTCT | GATGTTATGCTCTGCCTG | | OsP1g00110 |
| *RNA polymerase subunit alpha* | *Rpoα* | GCGTCTTTATTATGGTCG | TGTTCCTTCTGTTTCTCC | | Osplg00660 |
| *RNA polymerase subunit beta* | *Rpoβ* | GTGGGGAACTTGCTTTAGG | GCTTGTTGTATCCGTCTGA | | Osplg00240 |
| *RNA polymerase subunit β'* | *Rpo**β'* | CATAGATTAGGCATACAGGC | AATAGCGGGAGATAGGAG | | Osplg00250 |
| *RNA polymerase subunit β''* | *Rpo**β''* | AAAAGAGGAGGCTCGTGC | GATGTTGGCTAAGTGATTGA | | Osplg00260 |
| *Hydroxylamine reductase* | *NADH2* | ATCACTGTAGGACTTGGGTT | TTTCCAGAAGAAGATGCC | | OsPlg00810 |
| *NADH-ubiquinone oxidoreductase chain 4* | *NADH4* | TCCTTATTGCTTATGCTGTC | CCGTATGCTCCCATCTTTA | | OsPlg00910 |
| *ATP synthase subunit β* | *ATP**β* | TTATTGGACCCGTGCTGG | TTGCTTACCGTCAGTGTCTCG | | Osplg00410 |
| *Rubisco activase* | *RCA* | CTCTTCGTGCCCGTGTTTAC | TCGGAGTTAGCGTCACCAAG | | Os11g47970 |
| *Ribulose-bisphosphate carboxylase large subunit* | *RbcL* | CAACTGTTTGGACTGATG | GTTACCCACAATGGAAGT | | OsP1g00420 |
| *Ubiquitin* | *UBQ* | GCTCCGTGGCGGTATCAT | CGGCAGTTGACAGCCCTAG | | Os03g0234200 |

**Supplementary Table 4** Segregation for green and zebra plants in F_2_ populations from two crosses.

| F_2_ populations | No. of green plants | No. of zebra plants | Ratio | χ23:1 ^a^ |
| --- | --- | --- | --- | --- |
| *zl16*/Wuyunjing 7 | 436 | 134 | 3.25:1 | 0.599 |
| *zl16*/02428 | 120 | 37 | 3.24:1 | 0.104 |

^a^ Value for significance at *P* = 0.05 and 1 *df* is 3.84.

**Supplementary Table 5** Candidate gene prediction within the 58.2-kb region.

| No. | MSU Locus ID | Putative functions |
| --- | --- | --- |
| *ORF1* | *LOC_Os08g12760* | YT521-B,putative,expressed |
| *ORF2* | *LOC_Os08g12780* | Chloroplast envelope membrane protein,putative,expressed |
| *ORF3* | *LOC_Os08g12790* | Oytokinin inducible protein,putative,expressed/TE-related gene |
| *ORF4* | *LOC_Os08g12800* | Glucon endo-1,3-beta-glucosidose |
| *ORF5* | *LOC_Os08g12820* | Proteasome/cyclosome repeat containing protein,expressed |
| *ORF6* | *LOC_Os08g12830* | Cytidyly/transferase domain containing protein,expressed |
| *ORF7* | *LOC_Os08g12840* | FabA-like domain containing protein,expressed |
| *ORF8* | *LOC_Os08g12850* | Pentatricopeptide, putative, expressed |
| *ORF9* | *LOC_Os08g12890* | DNA binding protein, putative, expressed |
